# Evolution of cellular architecture and function of the hippocampus: insights from the artificial selection experiment

**DOI:** 10.1098/rsbl.2024.0617

**Published:** 2025-04-02

**Authors:** Anna Goncerzewicz, Elzbieta Bonda-Ostaszewska, Marcin Lipiec, Ewelina Knapska, Marek Konarzewski

**Affiliations:** ^1^ Centre of Excellence for Neural Plasticity and Brain Disorders, Nencki Institute of Experimental Biology, Polish Academy of Sciences, Poland; ^2^ Faculty of Biology, University of Białystok, Poland

**Keywords:** encephalization, hippocampus, trade-offs, experimental evolution

## Abstract

Inter-specifically, mammalian species with larger brains built of numerous neurons have higher cognitive abilities (CA) but at the expense of higher metabolic costs. It is unclear, however, how this pattern emerged since evolutionary mechanisms act intra-specifically, not inter-specifically. Here, we tested the existence of the above pattern at the species level in the hippocampus—the brain structure underlying CA. We used an artificial selection experiment consisting of lines of laboratory mice divergently selected for basal metabolic rate (BMR)—a trait implicated in brain size evolution, its metabolic costs and CA. Selection on BMR did not affect hippocampus size as a correlated response to this selection. However, the high BMR mice had superior CA and manifested increased neuronal density, higher cytochrome c oxidase density (indexing metabolic costs of neuronal activity) and dendritic spine density (indexing connectivity between neurons). Thus, our study calls into question the generality of patterns of the evolution of CA apparent interspecifically. At the species level, increased CA may arise through the rearrangement of the architecture and function of neurons without a conspicuous increase in their size but increase metabolism.

## 1. Introduction

Larger brains are considered a hallmark of increased cognitive abilities (CA; i.e. reasoning ability and behavioral flexibility, thereafter CA, e.g. [1,2]). Across species, the size (mass) of the mammalian brain varies by 100 000-fold, ranging from 0.04 g in Etruscan shrew [3] to 10 kg in sperm whale [4] (see also [5,6]). However, the CA of mammals do not vary to the same extent [7,8], which poses a question regarding the reasons for only a partial positive association between the size of the brain and CA [9,10].

At the interspecific level, the key pattern underlying increased CA of larger brains is an increased number of neurons that, in turn, are larger than those building smaller brains [11–15]. The pattern of a parallel increase in neuronal size and number in larger, more cognitively capable brains is apparent in major lineages [16]. A still unanswered question, however, is how this pattern has emerged, considering the primary constraint on the evolution of brain size and its architecture: the metabolic costs of maintaining brain tissue [17–20]. Indeed, brain energy expenditures account for *ca* 8% of basal metabolic rate (BMR) in mice but over 10% in primates and up to an exceptional 20% of BMR in humans, even though in all these species, the brain constitutes only 2–3% of body mass [21,22].

Equally intriguing is how the above interspecific patterns arose since evolutionary mechanisms act at the intra-specific, not interspecific level [23–25]. As rightly pointed out by [26], the most straightforward evolutionary mechanism would involve a positive selection of individuals having an above-average number of neurons of above-average size. If so, the allometric patterns of brain size and architecture discernible interspecifically should reflect the patterns detectable within species. Most importantly, they should also be reflected by the enhanced CA of 'brainier' individuals.

Here, we aimed to test the existence of the above congruity of inter- and intraspecific patterns of functional links between brain size, neuron size, metabolic expenditures and CA using the line types of laboratory mice subjected to artificial selection on body mass-corrected high or low BMR. Selection experiments, including ours, are an effective tool for investigating the linkages between traits of interest [27] while allowing for controlling other traits, such as body mass, which often confound interspecific comparisons [28]. In our experiment, divergent selection resulted in a 50% between-line type separation in BMR and a correlated differentiation in relative sizes (mass) of metabolically expensive internal organs—liver, heart and kidneys [20]. Yet, the high-BMR (H-BMR) mice did not have bigger brains than mice of the low-BMR (L-BMR) line type. Still, however, the H-BMR mice were superior in the reward-seeking and aversive cue discrimination learning tasks, indexing their high CA. Furthermore, the H-BMR mice had higher plasticity of hippocampal neurons (indexed as long-term potentiation, LTP) than the animals from the (L-BMR) line type. Thus, our previous results indicate that higher CA and underlying neuronal plasticity can be realized in a mammalian brain without increasing its size, as apparent at the interspecific level.

Here, we explore this apparent conundrum, concentrating on the size and cell architecture of the hippocampus—a brain structure critically involved in forming, organizing and retrieving new memories [29–31]. We hypothesize that the differences between line types in hippocampal size and structure most likely underlie the respective differences in CA (i.e. performance in the reward-seeking or aversive cue discrimination learning tasks) reported by [20]. As the number and size of neurons are not the only determinants of brain information-processing capacity, we also analysed the number of dendritic spines in key hippocampus areas as a proxy for neuronal connectivity [32–34]. Finally, we analysed the differences in the density of cytochrome c oxidase (CCO) in the hippocampus to estimate the metabolic costs of its activity [35].

## 2. Material and methods

### (a) Animals

We used 3- to 4-month-old Swiss–Webster female mice from the selection experiment at the Faculty of Biology, University of Bialystok, Poland. We divergently selected mice of the H-BMR and L-BMR line types for high/low body mass-corrected BMR quantified according to the procedure detailed elsewhere [36,37]. As we demonstrated earlier [20], the H-BMR and L-BMR mice differ not only concerning BMR but also in terms of CA, evaluated as performance in the reward-seeking and aversive cue discrimination learning tasks (for more information, see electronic supplementary material).

We also used female Swiss–Webster mice from four random bred (RB) lines maintained concurrently with the selection on BMR (for details, see [20]). Mice of these lines formed the RB line type, referencing the divergence of traits analysed therein in animals selected for BMR. For information on the number of animals, see electronic supplementary material, table S1.

### (b) Hippocampus size, hippocampal pyramidal area and neuronal density

For measurements of hippocampus size and hippocampal pyramidal area, we used transverse (coronal) cross-sections of the hippocampus positioned −1.46, −1.82, −2.3 and −2.7 mm from the intersection of the https://en.wikipedia.org/wiki/Coronal_suture (i.e. from bregma, coded as the effect of position, with *n* = 4 for each animal), mounted on gelatin-coated slides and stained according to the standard Nissl method. Microscope images were captured using a light microscope, Leica DM1000 LED, connected to a Leica ICC50 camera. The total surface area (µm^2^) of each of the cross-sections of the hippocampus (being a proxy of its size for a given animal), as well as the pyramidal cell layers area, were traced manually and then measured at each position using CellSens Dimension Desktop (Olympus Corp., Japan).

Neuronal density was estimated by a single rater in the pyramidal cell layer in the CA1, CA2 and CA3 regions of the left and right sides of the hippocampus (thereafter lateralization) in the same four two-dimensional cross-sections as used for the estimation of the area occupied by the pyramidal cell layer. Neurons were counted inside rectangular frames of 3000 μm^2^ (counting frame) localized at random in the central part of each of the CA1−CA3 regions (electronic supplementary material, figure S1). In total, four frames (each representing one position) were analysed for each hippocampal region, and then measurements were averaged within a given region. Thus, we estimated neuronal density in 3 (regions) × 2 (lateralization) × 4 (position) = 24 samples per animal. For details, see electronic supplementary material.

### (c) Cytochrome c oxidase activity staining

The histochemical reaction for CCO was prepared according to the published protocol [38]. For details, see electronic supplementary material.

### (d) Dendritic spine density analysis

Because of logistic limitations, the dendritic spine density (DSD) analysis was restricted to only the H-BMR and L-BMR mice (five animals from each line type, see electronic supplementary material, figure S3). Dendritic spines were visualized using the lipophilic dye Dil (1,1′-dioctadecyl-3,3,3′,3′-tetramethylindocarbocyanine perchlorate, no. D282 Life Technologies, Warsaw, Poland). The dye was delivered to cells using the Gene Gun (Bio-Rad). Z-stacks of dendrites from the CA1, CA3 and the DG regions of the hippocampus were acquired using a Zeiss LSM 880 confocal microscope with AiryScan on super-resolution mode, using a 63× magnifying lens for high-resolution imaging (Plan Apochromat 63×/1.4, Zeiss). For details, see electronic supplementary material.

There were no clearly stained dendrites in the CA2 region, so they were excluded from further analyses.

### (e) Statistical analyses

We log10-transformed dendritic spine and CCO density measurements because of the strong right-skewness of the distribution. The data are available from the Dryad Digital Repository [39].

Measurements of hippocampal size and pyramidal cell area were analysed using ANOVA (Mixed procedure of SAS/STAT® 14.1, SAS [40]) with line type affiliation (RB, H-BMR or L-BMR line type), positioning of cross-sections with respect to bregma (position) and lateralization as fixed factors and their respective interactions. Replicated lines nested within line types were coded as the random factor of the model (four replicated lines in the RB line type, but one line for the H-BMR and L-BMR line types, respectively, as they were not replicated; six lines in total). The respective mean squared error for six lines was used as the denominator of the F statistics testing the effect of line affiliation. Hence, the d.f. for the between-line type comparisons was 2 (for the F numerator) and 3 (for the denominator). Likewise, the d.f.for planned pairwise *t*‐test comparisons between the line types was 3.

Neuronal density estimation and CCO were analysed using ANOVA as described above, with the additional fixed factor, region, coding for CA1–CA3 hippocampal regions.

DSD was analysed using ANOVA with line type affiliation and the hippocampal region as fixed factors and the respective interaction.

Our statistical models initially included all respective interactions. The models were then step-wise reduced by removing non-significant interactions (*p* > 0.05). All initial analyses also included lateralization and, whenever applicable, the generation of origin (coded as fixed factors). Both lateralization and generation effects were never significant (*p* > 0.05) and, therefore, dropped from the final analyses.

### (f) Evaluation of the effect of genetic drift on dendritic spine density

The data set on DSD consisted of the measurements carried out on mice from the L-BMR and H-BMR lines but not the RB mice. Thus, unlike other comparisons between the L-BMR and H-BMR mice versus four RB lines, this data set does not allow for an unequivocal exclusion of the effect of genetic drift as a potential source of the observed between-line differences in DSD. We therefore compared the magnitudes of the between-line type separation in DSD with those expected under genetic drift according to the guidelines worked out by [41,42]. For a detailed description of this analysis, see electronic supplementary material.

## 3. Results

### (a) Hippocampus size and neuronal density

The total cross-sectional surface area of the hippocampus (being a proxy of its size) did not differ between the line types (i.e. H-BMR and L-BMR), along with the RB reference line type (F_2,3_ = 0.01; *p* = 0.99). The effect of the position of the cross-section with respect to bregma (i.e. position effect) was statistically significant (F_3,129_ = 70.65; *p* < 0.0001), in contrast to the line type × position interaction (F_6,129_ = 0.82; *p* = 0.6).

The line type effect on pyramidal hippocampal cell layer area was not significant (F_2,3_ = 0.03; *p* = 0.97), albeit with a significant effect of the position of the cross-section (F_3,130_ = 13.5; *p* < 0.0001) and a significant line type × position interaction (F_6,130_ = 3.21; *p* = 0.006). Further analyses carried out separately for each position did not reveal a significant effect of the line type (*p* > 0.1, in all cases). Overall, our results suggest that the selection on BMR did not affect the structural size of the hippocampus.

**Figure 1.**
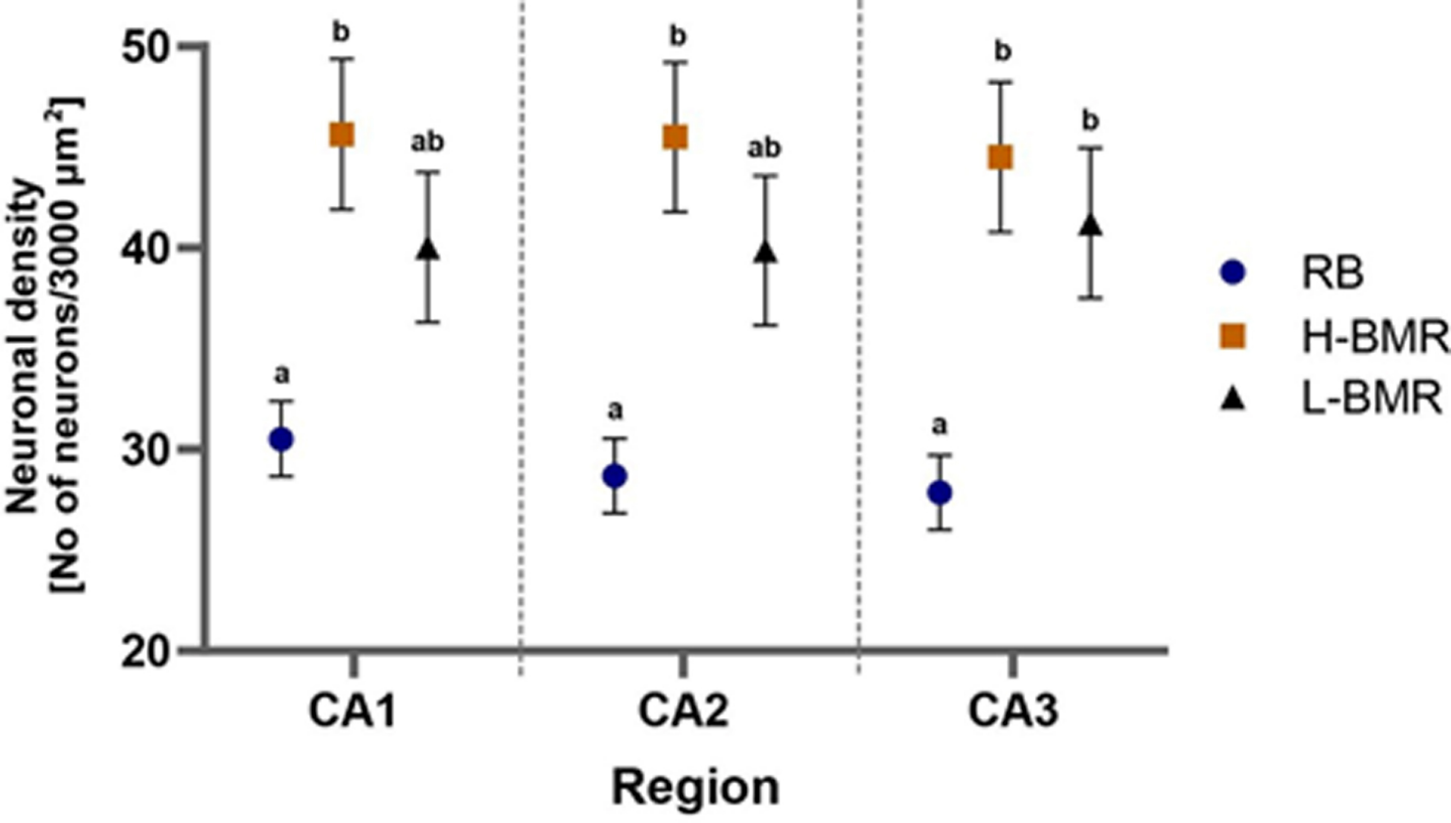
Neuronal density in the CA1, CA2 and CA3 regions of the pyramidal layer of the hippocampus was obtained using Nissl staining. Points indicate least square means (± SE) derived from the ANOVA for mice from the H-BMR, L-BMR and RB line types. When labelled with different letters, the means differed from each other at *p* = 0.05.

Neuronal density in the pyramidal hippocampal cell layer differed significantly between the line types and hippocampal regions (thereafter the region effect; table 1, figure 1). Planned pairwise comparisons revealed that the neuronal density of the H-BMR line type was higher than that of the RB line type in all (CA1–CA3) regions.

**Table 1.** ANOVA results for the hippocampus anatomical and cellular characteristics. In all comparisons, the effects of lateralization and position were insignificant and thus, dropped from the final models.

	line type	region	line type × **region**
neuronal density^a^	F_2,3_ = 9.66 *p* = 0.05	F_2,396_ = 1.83 *p* = 0.2	F_4,396_ = 3.74 *p* = 0.005
neuronal density^b^	F_2,7_ = 6.94 *p* = 0.02	F_1,109_ = 228.4 *p* = 0.001	F_2,109_ = 6.29 *p* = 0.003
astrocyte/neuron surface ratio	F_2,3_ = 9.40 *p* = 0.05	F_2,111_ = 5.52 *p* = 0.005	F_4,111_ = 3.90 *p* = 0.005
dendritic spine^c^ density	F_1,226_ = 2.79 *p* = 0.09	F_2,226_ = 4.36 *p* = 0.01	F_2,226_ = 6.63 *p* = 0.002
cytochrome c oxidase	F_2,3_ = 6.20 *p* = 0.09	F_3,254_ = 147.7 *p* < 0.0001	F_6,254_ = 47.4 p = < 0.0001

^a^ Nissl staining

^b^ DAPI staining

^c^ This comparison was carried out between the H-BMR and L-BMR line types.

### (b) Dendritic spine density

**Figure 2.**
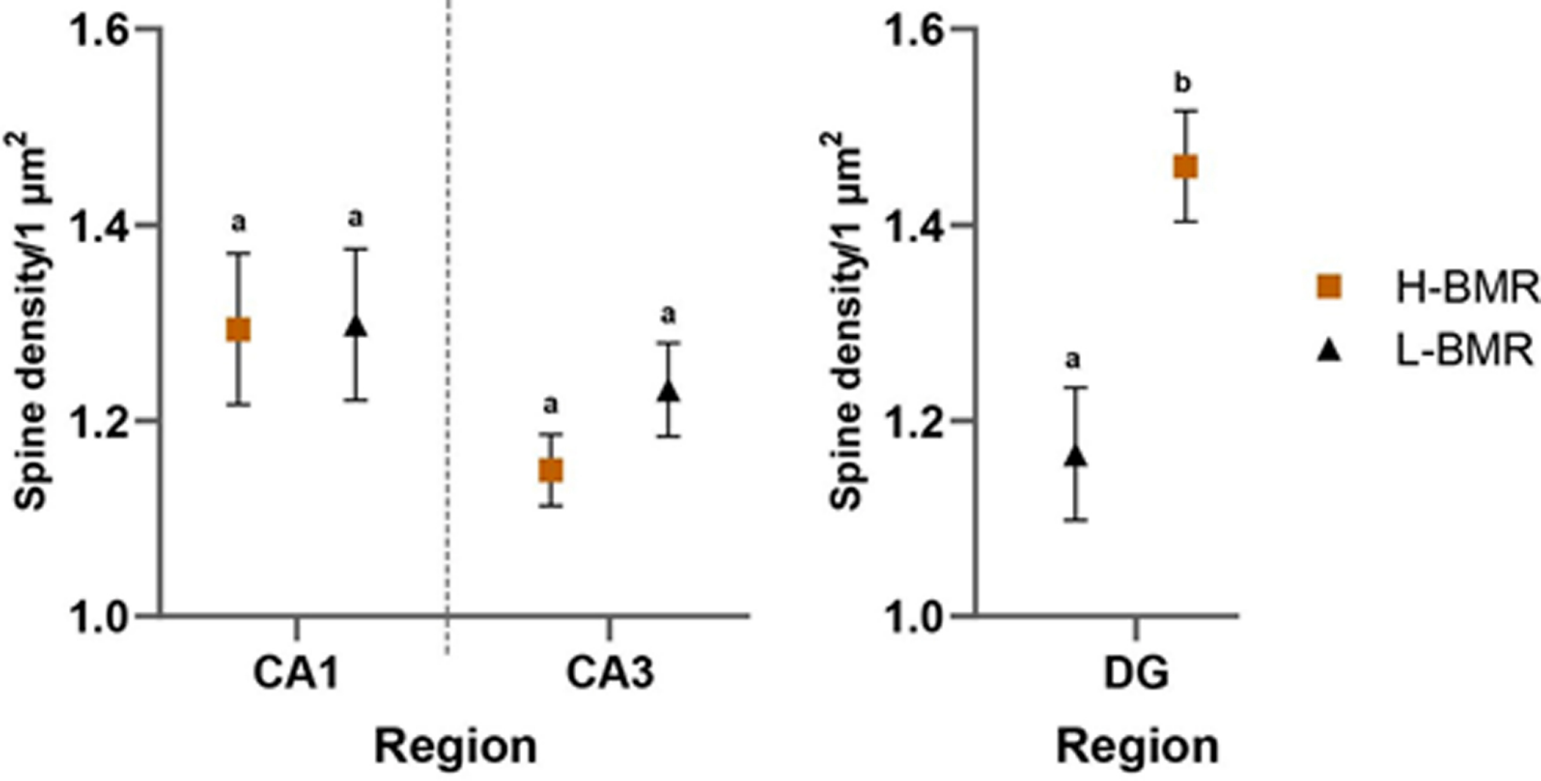
Dendritic spine density in the CA1, CA3 and DG regions of the hippocampus is shown. Points indicate least square means (± SE) derived from the ANOVA. When labelled with different letters, the means differed at *p* = 0.05. Slices from the H-BMR (orange squares, *n* = 5) and L-BMR (black triangle, *n* = 5).

DSD, a proxy of neuronal connectivity, was significantly affected by the line type affiliation and the region effect, as indicated by its highly significant interaction (table 1). This interaction, however, was not due to the difference observed in the CA1 and CA3 regions but because DSD was significantly higher in the dentate gyrus (DG) region of the H-BMR mice compared with the L-BMR line type (figure 2). This result is significant as the DG is the input region of the hippocampus, playing a critical role in learning, memory and spatial coding. The resulting divergence in DSD in the DG is sufficiently large to be confidently attributed to the applied selection rather than to genetic drift (see electronic supplementary material, figure S4, for a detailed description of the analysis).

### (c) Cytochrome c oxidase density

**Figure 3.**
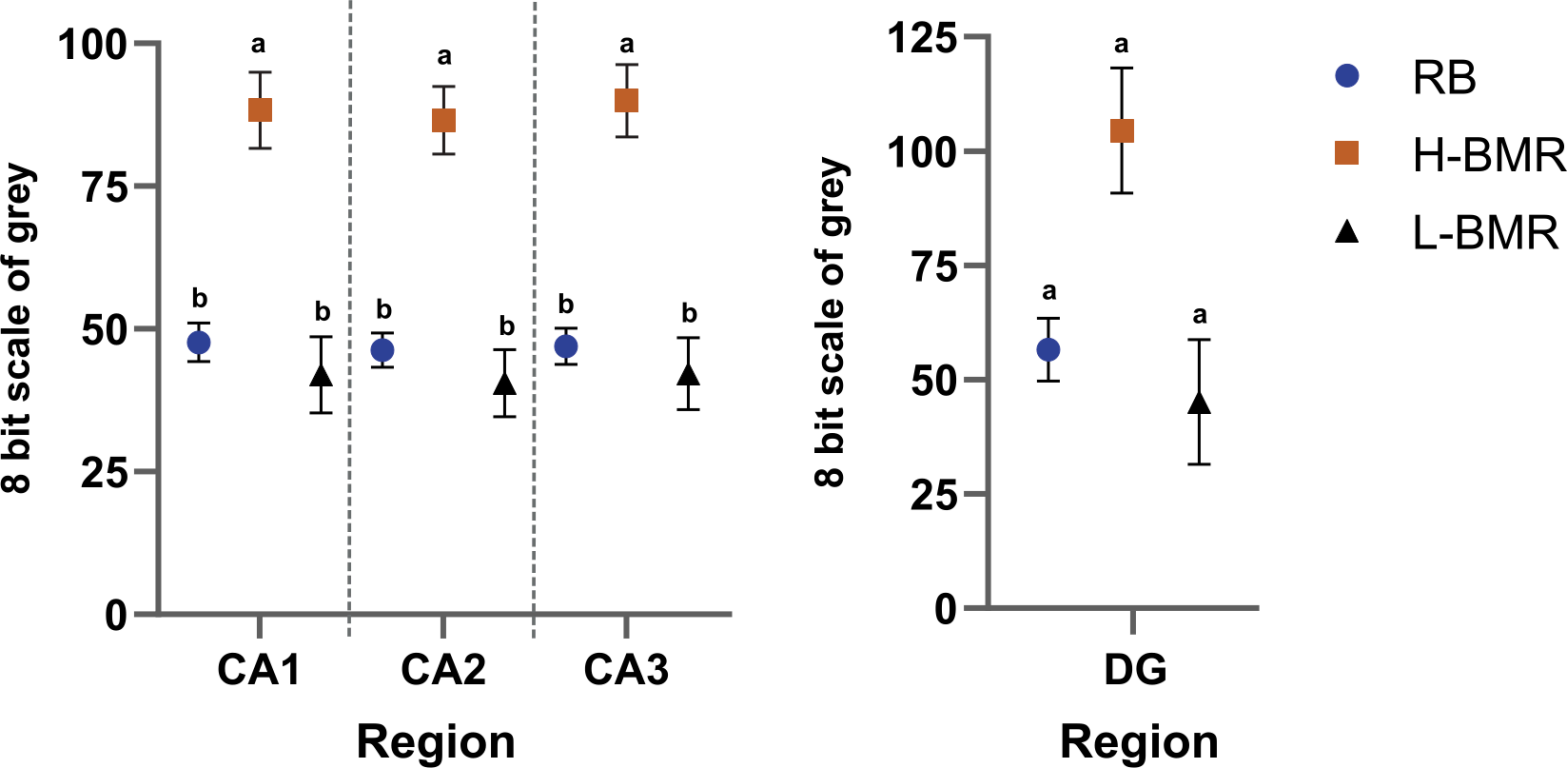
CCO density in the CA1, CA2, CA3 and DG regions of the hippocampus. The scale represents the intensity of the colour in an 8-bit scale of grey. The intensity was subtracted from 255—the maximum value of the scale—to transpose the inverse relation between the intensity of the colour and CCO density. When labelled with different letters, means (i.e. least square means (± SE) derived from the ANOVA) for the H-BMR line type differed at *p* = 0.05 from the other line types (by *a priori* custom-made contrasts).

CCO density (a proxy of mitochondrial density) significantly varied between the line types and regions, with a significant line type × region interaction (table 1). The line type effect was clearly due to CCO density being much higher in the H-BMR line type than in the other mice (figure 3).

## 4. Discussion

Our results revealed that the H-BMR, L-BMR and RB mice differed statistically significantly in several key parameters of the hippocampal cell architecture and function. In particular, we found that the H-BMR mice, characterized by superior performance in the reward-seeking and aversive cue discrimination learning tasks, indexing CA [20], manifested increased neuronal density contributing to the neural basis of CA, higher CCO density (indexing metabolic costs of neuronal activity) and DSD (indexing connectivity between neurons). However, in most cases, statistical significance was at best on the verge of the commonly adopted *p* = 0.05 level (table 1). Therefore, it is worth highlighting that our statistical inference is based on only 3 d.f., constituted by the respective line types. Importantly, if we considered the numbers of individual mice as d.f. (as in, e.g. [26,43]), our comparison of the neuronal density would be significant at an impressive but erroneous *p* < 0.001 (with F_2,17_ = 45.6), rather than at the correct, but unimpressive *p* = 0.05 (table 1). For the same reason, even though the effect of the position × line type interaction on the pyramidal hippocampal cell layer area was statistically significant at *p* = 0.006 (see ‘Results’), the analyses carried out separately for each position did not reveal a significant effect of the line type (*p* > 0.1, in all cases).

Our statistical approach is the only proper way to avoid pseudoreplication and allow for an appropriate distinction of the effects of selection from random effects, such as genetic drift [42]. Thus, we are confident that our statistical inference is robust despite the apparently low levels of statistical significance of the reported tests. For the same reason, the lack of the between-line type differences in the hippocampus size found therein is unlikely to be prone to type I statistical error.

Overall, we detected several between-line type differences likely to account for higher CA demonstrated by the H-BMR mice in cognitive tests reported by [20]. First, their neuronal density in the hippocampus was higher than that of the RB animals (table 1, figures 1 and 2), which served as a reference in [20]. As the size of the hippocampus did not differ between the line types, higher neuronal density translates to more numerous neurons, which is generally associated with higher CA [14]. Second, DSD was significantly higher in the DG region of the H-BMR mice, compared with the L-BMR line type, which was inferior in the cognitive tests [20].

Dendritic spines provide connectivity between neurons and undergo changes in their number and morphology driven by external stimuli [44]. The DG is the predominant entry point for synaptic input to the hippocampus [45]. Therefore, the density of spines in this region is likely to be tightly associated with fast learning and memorization [30]. The above between-line differences form the cellular foundation of the positive genetic correlation between CA and BMR, as reported by [20].

We must admit, however, that our results revealed a couple of conspicuous inconsistencies that are nevertheless informative. First, although the H-BMR mice manifested significantly higher neuronal density than the RB (control) animals, pairwise comparisons indicated that its level did not statistically differ from that found in the L-BMR mice (figure 1). Therefore, the trend apparent in figure 1 suggests that divergent selection on BMR did not result in a parallel divergence in neuronal size between the high and low BMR line types. Notably, the lack of this divergence was paralleled by the lack of the between-line type difference in hippocampal size. Thus, higher CA of the H-BMR mice compared to that in the L-BMR animals is unlikely to be solely associated with more numerous neurons. This agrees with the results reported by [43], who did not observe significant correlations between performance in behavioural tests and the number of neurons in the hippocampus of Swiss mice.

Furthermore, Goncerzewicz *et al*. [20] demonstrated higher LTP of neurons in the CA1 region of the hippocampus in the H-BMR than in the L-BMR mice. LTP is considered a proxy of central cellular mechanisms of learning and memory formation [46]. However, the present study did not find a between-line type difference in DSD in the CA1 region, even though spine formation and re-arrangement are considered the key components of memorization [30]. It is possible, however, that the lack of differences in DSD of the CA1 region is compensated by the higher spine density in the DG region (figure 2).

Generally, our results show that the differences in CA may arise without a conspicuous differentiation in hippocampal size, as reported in humans [47,48] and non-humans [49,50]. It, therefore, raises the question of why our selection resulted in the differentiation of CA without a straightforward scaling up of the hippocampus and, more generally, the whole brain, as observed interspecifically. This question is even more puzzling when one considers that the evolutionary ‘solution’ identified here in the H-BMR line type came at a substantial energetic cost, as indicated by a high density of CCO in the CA1–CA3 hippocampal regions (figure 3).

The answer to the above question is the existence of a positive association between BMR and the metabolic costs of maintaining the brain tissue. Indeed, such an association was demonstrated in mammals at the interspecific level [17]. In rodents, as much as 20% of interspecific variation in body mass-corrected BMR can be attributed to brain mass variation [51]. As BMR reflects the maintenance costs of metabolically expensive organs [52], it must also include the metabolic costs of, e.g. synaptogenesis [22,53]. As we pointed out in the Introduction, however, to be effective as an evolutionary mechanism driving encephalization, the BMR–brain size association must be present at the species level, where natural selection operates [23–25]. Our results and Goncerzewicz *et al*.’s [20] study show that, at least in our animal model, the positive genetic correlation between BMR and brain size is absent. However, divergent selection for BMR resulted in a correlated response of increased CA in the H-BMR mice [20]. Thus, enhanced CA was indeed associated with high BMR, reflecting increased neuronal metabolism, as suggested by increased CCO density, but not an increased brain size.

Our findings demonstrated associations that run counter to interspecific patterns widely considered as mechanisms of the evolution of CA, in particular, the nexus of larger neurons and larger brains [11–15]. To our knowledge, at the intra-specific level, this nexus has been reported only for a small fish—the guppy (*Poecilia reticulata*) [54,55]. Contrasting results reported by [55] and herein are of particular importance because both studies present inferences from models of experimental evolution, which are much more robust than those of [26] and [43], which are based on an analysis of uncontrolled phenotypic variation [27]. The life history and physiology of fish are far removed from that of homeotherms [56,57]. Therefore, its relationship with mechanisms of selection on encephalization in mammals is unclear. Moreover, a recent interspecific analysis carried out by [16] indicated that mammals do not follow the pattern of a significant positive correlation between relative brain size and neuronal density, except for primates. Yet, these findings disagree with an earlier study by [58] showing that rodents also comply with the large brain—large neurons nexus.

Our study supports the conclusions drawn by [16] and provides the first experimental evidence demonstrating that a substantial differentiation of CA in non-primate mammals could have originated without a parallel increase in brain and neuron size. Thus, an evolutionary increase in BMR does not relax constraints on their growth. Finally, our results suggest that the key cost of enhanced CA takes the form of increased neuronal energy consumption, as indicated by high CCO density.

## Data Availability

All raw data will be available from the Dryad Digital Repository [59]. Supplementary material is available online [60].
